# Why interventions to prevent intimate partner violence and HIV have failed young women in southern Africa

**DOI:** 10.1002/jia2.25380

**Published:** 2019-08-23

**Authors:** Jenevieve Mannell, Samantha Willan, Maryam Shahmanesh, Janet Seeley, Lorraine Sherr, Andrew Gibbs

**Affiliations:** ^1^ Institute for Global Health University College London London UK; ^2^ Gender and Health Research Unit South African Medical Research Council Pretoria South Africa; ^3^ Africa Health Research Institute KwaZulu‐Natal South Africa; ^4^ Department of Global Health and Development London School of Hygiene and Tropical Medicine London UK; ^5^ Centre for Rural Health School of Nursing and Public Health University of KwaZulu‐Natal Durban South Africa

**Keywords:** HIV, IPV, adolescents, youth, southern Africa

## Abstract

**Introduction:**

Adolescent girls and young women aged 15 to 24 years have some of the highest HIV incidence rates globally, with girls two to four times more likely to be living with HIV than their male peers. High levels of intimate partner violence (IPV) experienced by this age group is a significant risk factor for HIV acquisition. While behavioural interventions to prevent IPV and HIV in southern Africa have seen some success in reducing self‐reported experiences of IPV, these interventions have largely failed to achieve similar outcomes for young women.

**Discussion:**

We identify three main reasons for the failure of IPV/HIV interventions for many young women in southern Africa. First, interventions are usually developed without the meaningful involvement of both young women and young men. Youth input into research design is largely focused on user testing or consultation of targeted groups, involving relatively low levels of participation. Second, interventions are focused on addressing individual risk factors rather than broader social and structural contexts of being a young woman. “Risk factor” interventions, rather than supporting women's agency, can pose a major barrier for supporting changes in behaviour among young women because they often fail to dislodge well‐entrenched gender and age‐related inequalities. Third, current intervention models have not adequately accounted for changes in gender norms and relationships across southern Africa. Individuals are getting married later in life (or not at all), new technologies are transforming romantic interactions and opening new opportunities for violence, and discussions about women's rights are both challenging gender inequalities and reinforcing them.

**Conclusions:**

In order to move beyond the status quo of current approaches, and to support real innovation, IPV/HIV prevention interventions need to be co‐developed with youth as part of a meaningful participatory process of research, intervention design, youth involvement in development and implementation. This process of co‐development needs to be radical and break with the current focus on adapting existing interventions to meet the needs of young people, which are not well understood and often do not directly reflect their priorities. Broader social contexts and compound lenses are needed to avoid narrow approaches and to accommodate evolving norms.

**Abbreviations**
HIV: human immunodeficiency virus
IPV: intimate partner violence
PrEP: pre‐exposure prophylaxis
SSCF: stepping stones creating futures

## 1 Introduction

Adolescent girls and young women aged 15 to 24 years, particularly those out of school, have some of the highest HIV incidence rates globally, with girls two to four times more likely to be living with HIV than their male peers 1. High levels of intimate partner violence (IPV) experienced by this age group is a significant risk factor for HIV acquisition 2, 3. IPV places young women at greater risk of depression 4, anxiety 5, and harmful alcohol use 6, which in turn increases their risk of HIV 7, 8. While IPV increases the susceptibility of young women to forced sex by male partners 9, far more problematic and insidious are connections between men's use of violence against female partners and men's attempts to control women's autonomy and assert male power, reducing young women's self‐efficacy and ability to negotiate safe sex 10, 11.

Young women's susceptibility to HIV is linked closely to their male partner's risk behaviours. Men who are violent tend to have other high‐risk behaviours including substance abuse, and multiple and concurrent sexual partners, and are more likely to be living with HIV 12, 13. IPV often belongs to a wider context of violence for young men, including witnessing violence within their household or in broader society as children, making them more likely to perpetrate violence as young adults 14. Exposure to violence for both adolescent boys and girls contributes to lower academic performance in school 15 and internalized stigma among youth living with HIV 16, further contributing to a cycle of violence and poverty that reduces individual opportunities and increases HIV risk.

While behavioural interventions to prevent IPV and HIV in southern Africa have seen some success in reducing self‐reported IPV experience and perpetration, and HIV risk behaviours, these interventions have largely failed to achieve similar outcomes for young women 17, 18, 19, 20, 21, 22. For instance economic interventions for women – particularly when combined with gender transformative interventions – have shown promise at reducing IPV and HIV risk, but three reviews have shown that these positive outcomes have typically been amongst older women, in more stable (often rural) settings, and not among adolescents 17, 18, 22. While a recent systematic review of HIV prevention interventions highlighted the lack of consistent positive findings by evaluations among young women 23. This is compounded, by the fact that there is little age‐disaggregated information within evaluations; a systematic review of adolescent focused IPV and HIV prevention interventions found only six rigorous evaluations, of which only one showed reductions in physical IPV for young women in school, another showed an impact on young men's perpetration of IPV, and the other four, did not disaggregate by age 24.

These failures have knock‐on effects for HIV‐related biomedical interventions with adolescent girls and young women. Reviewing oral antiretroviral pre‐exposure prophylaxis (PrEP) for young women in sub‐Saharan Africa, Celum and colleagues 25 point to the limited overall impact among women under the age of 21 linked primarily to poor engagement and adherence. They argue the failure of PrEP interventions for young women is the result of the lack of consideration of local contextual factors, including social norms around sexuality and broader structural barriers making it difficult for young women to adhere to PrEP. Similar findings of low adherence in younger women were described in the topical microbicide and vaginal ring trials 26, 27.

A radical new approach to behavioural interventions that prevent HIV and IPV is urgently needed to address the context‐specific needs of young women living in southern Africa, including issues of economic and relationship insecurity and the implications these have for HIV prevention. In this commentary, we discuss some reasons behind the failure of behavioural IPV and HIV risk prevention interventions for young women with the purpose of identifying a potential way forward for future intervention development.

## 2 Discussion

Through our collective experience working on behavioural interventions to prevent IPV and reduce HIV risk across sub‐Saharan Africa (see Table 1), and engagement with the reviews described above, we have identified three main reasons for the failure of these interventions for many young women. First, interventions are usually developed without the meaningful involvement of both young women and young men. Second, interventions are largely focused on addressing individual risk factors rather than supporting spaces for young women's agency. Third, current intervention models have not adequately accounted for changes in gender norms and relationships across southern Africa. We outline each of these reasons in turn and discuss how they contribute to failed interventions.

**Table 1:** Behavioural interventions to prevent IPV and reduce HIV‐risk by commentary authors

Author(s)	Study title	Objective	Outcomes	References
AG; SW	Pilot and randomized control trial of the Stepping Stones and Creating Futures intervention	Reduce IPV experience, and HIV risk behaviours amongst young women in urban informal settlements in South Africa	No impacts on HIV risk or IPV experience, but improved livelihoods	28, 29, 30
AG	Strengthening community responses to HIV in rural South Africa	Strengthen local community involvement in the HIV response, including young people's engagement	Young people felt excluded from HIV spaces because of adult power	31, 32
SW; AG	Applied Research Services on Inter‐Linkages Between Gender Based Violence and HIV	To strengthen guidance for local organizations on integrating IPV prevention into HIV programming	N/A this was a guidance document	33
MS; JS; LS	Determined Resilient Empowered AIDS free and Safe impact evaluation	To evaluate the impact of the DREAMS combination community, family and individual intervention on HIV incidence in young women in rural KwaZulu‐Natal	Scaling up complex interventions is feasible, however, reaching out of school and mobile young women is challenging.	34
MS; JS	Thetha Nami: Co‐Creating peer‐led interventions to support uptake and retention in multi‐level HIV care and prevention	To work with area‐based teams of young men and women to optimize the delivery of multi‐level HIV prevention and care including adapting biomedical innovations	Young people have a nuanced understanding of the complexity of their context and are able to optimize and deliver innovative area‐based intervention that include adaptive use of newer technologies such as HIV self‐testing and community‐based Pre‐Exposure Prophylaxis.	35
LS	Community Care Study	Examining the impact of Community‐based organization support on those infected and affected by HIV	CBO provision positively impacted youth and provided an understanding of violence, mental health, development and cash transfers	36, 37
JM	Community responses to intimate partner violence	To conceptualize agency and community capacity in responding to intimate partner violence in Rwanda	Agency of young women is multifaceted and “distributed” across time, space and social location	38, 39, 40

### 2.1 The absence of meaningful involvement of young people in designing interventions

Innovation in interventions with the aim of meeting the needs of young women requires the direct involvement of young women and men in the design of interventions, however, this often does not happen in a meaningful way. Where there is input in intervention design, it is largely focused on user testing or consultation of targeted groups, involving relatively low levels of participation. For example in *Stepping Stones and Creating Futures (SSCF),* which involved two of the authors (AG & SW) *–* a curriculum‐based intervention to reduce IPV and HIV among young people in urban informal settlements in South Africa *–* the livelihoods manual (Creating Futures) was developed using a log‐frame, and then tested with 20 young men and 20 young women through a five‐day workshop. On the basis of the workshops, the manual was revised tested again with new groups, and finalized before piloting the intervention 41. While this form of involvement of young people in the design of interventions is a strong move in the right direction and is a dominant approach in programmatic design 42, it is a far cry from the meaningful involvement of young people as active participants in the research process as a means of bringing local perspectives into the way research itself is conceptualized and how it informs intervention design 43.

New approaches to intervention development such as “human centred design” offer potential approaches which centralize participants’ experiences and perspectives 44. However, in a recent review of human centred design in global health, few examples went beyond undertaking focus groups and some basic ethnographic research, to meaningfully involve those who the intervention is designed for in the development, piloting and refining of the intervention 45. Engagement needs to go beyond simplistic incorporation of “users” views.

The lack of meaningful involvement of key populations in the development or adaptation of interventions contributes to interventions not resonating with the current priorities of young people. For example the implementation of IPV prevention interventions targeting adolescent girls and young women in rural, deprived areas of KwaZulu‐Natal, South Africa, with persistently high HIV incidence illustrated challenges of scaling up curriculum‐based interventions 46. One key challenge was poor uptake and completion rates, particularly by those young women out of school or who reported recent migration. While young women welcomed the focus on their needs and the opportunity to learn about health, they described a disconnect between the focus of the programme (individual‐level risks that they did not always identify with), in contrast with the community‐wide risks and anxieties they faced. Their anxieties related to livelihoods, lack of opportunities, hope and even recreation, and significant worries about future fertility and ability to bear children. Adapting content to relate to the social context and needs of the young women and the delivery model to fit into their daily routines and life structure may help overcome some of these barriers to exposure and thus impact.

Without the meaningful involvement of young people in intervention implementation design, intervention delivery fails to adapt to the structural barriers of time, space and convenience that constrain the daily lives of young women. This limitation is evident in group‐based curriculum‐driven interventions, which involve sessions held at regular intervals across a number of weeks. While this mode of delivery is based on theories of adult learning, which assume learning happens through reflection and “testing” of strategies, with time between sessions allowing this to happen 47, in contexts where young women are out of school, mobile and with multiple competing priorities, adhering to regularly scheduled activities can be impractical. In low‐income settings, young women who are not in school are often involved in a repetitive process of seeking piece work, which may be sporadic, require long hours and be located long distances from home. Similarly, young unemployed women are often expected to care for any children in her extended family.

Meaningful involvement of young people in the development of interventions, including in how they are designed is critical for overcoming these challenges. By meaningful involvement we refer to approaches such as the co‐development of interventions, where those targeted by interventions are the ones who design interventions, with academics and practitioners supporting young people in this process 48. When sufficient time is allowed, young people can be supported to come to analyse their own, and their peers’, lives and identify strategies and intervention models that resonate with their own life worlds and experiences, rather than ones mediated by researchers.

### 2.2 Interventions are developed based on an analysis of risk factors

There is a strong evidence‐base outlining HIV risk factors for young women in southern Africa, including IPV 13, 49, transactional sex 50, multiple partners 51, alcohol and substance use 52, and poor mental health 7, 8. Interventions are often designed to reduce these risk factors, 53 for example to reduce transactional sex or risky behaviours, interventions focus on building young women's access to savings or reducing alcohol consumption.

An alternate approach has been to focus on “structural drivers” of HIV risk 54. These recognize that risk is not produced by the individual, but rather by contexts that determine the limits, and options, for specific practices/behaviours 54. Recognizing this is an important step away from individualized understandings of risk for HIV and IPV 55. However, ultimately, such approaches still aim to reduce risk, rather than a more radical approach to intervention conceptualization.

Interventions focused on tackling “risk factors” whether at the individual or structural level, rather than supporting women's agency, can pose a major barrier for supporting changes in behaviour among young women because they often fail to recognize how gender and age inequalities intersect. For example, interventions working on IPV and HIV prevention may fail to acknowledge the ways in which some women perceive violence as an acceptable aspect of a loving relationship 28, or how others respond to violence by protecting their abusers in order to avoid further discrimination from family and community members 38.

It also neglects the fact that sex is an activity that is often pleasurable and fun. Thus, a simple focus on where “risk” comes from fails to adequately understand the multiple factors shaping women's decision‐making and ability to change their lives.

Rather, we suggest there needs to be a radical shift away from “risk factor interventions” focused on individual risk behaviours and risk factors, towards identifying the spaces that exist for young women's agency to address these risks and supporting women's agency. This requires a shift in thinking about how interventions are developed to identify and strengthen what Campbell and Mannell refer to as “distributed agency,” essentially the range of agentic actions young women can take, even in contexts of oppressive relationships 39. If interventions to prevent IPV and HIV acquisition are aiming to “strengthen” agency, then an understanding of where young women can assert agency from their own perspectives (e.g. by manipulating a boyfriend to give them money but avoiding sexual encounters), needs to be central to the development of interventions rather than simply a focus on risks they face. Working to enhance women's own strategies, and mitigate any negative outcomes of these actions, must be central to the work of interventions 55.

### 2.3 Gender norms and relationships are rapidly changing in southern Africa

Gender norms and intimate relationships are not static social phenomena, but change over time 56. For example women are increasingly taking on traditionally male roles as the head of households (some by necessity and others by choice) coinciding with a number of young people not getting married or marrying much later in life 57. Discussions about women's rights have taken centre stage, often conflicting with notions of gender in ways that challenge gender norms but can also reinforce them 58. Conversations about same‐sex relationships and transgender rights are also happening, however, the specific needs of these groups are still being neglected in ways that exclude some of the most vulnerable young women and men from interventions 59.

Moreover, the use of new technologies including cellular phone use and social media has increased substantially across southern Africa 60, creating new spaces for health intervention and social interaction 61. However, this has also created new opportunities for gendered forms of violence to occur. For example in addition to offering new communication opportunities, the widespread penetration of cell phones in Zambia has provided opportunities for new forms of controlling behaviours by husbands and the justification of violence by boyfriends through monitoring women's cell phone use 62. These changes in gender norms and the advent of new technologies are increasing the complexity of choices available to young women in ways that affect potential IPV and HIV risk reduction interventions.

The interventions we are using to address IPV and HIV risk not only often fail to acknowledge these new manifestations of gender norms but may also reinforce old ones. Economic interventions that provide alternative livelihoods for women to support them in leaving violent relationships have often reinforced women's roles as homemakers through sewing or handicraft interventions 63. In addition, supporting women to work and have livelihoods is often in addition to women's other responsibilities, including unpaid care work and household labour. The gender norms that condone young women's financial dependence on men's productive work and women as unpaid labourers have remained largely unchallenged.

## 3 Conclusions

Addressing the three challenges of IPV and HIV interventions we have highlighted in this commentary requires a bold new approach. Specifically, behavioural interventions for young women to prevent IPV and HIV need to be co‐developed with youth as part of a long‐term participatory process of research, intervention design and implementation. This process of co‐development needs to be radical and break with the current focus on existing interventions to support real innovation. It will require that academics and practitioners take a step back and reorient their role from designing interventions, into one of supporting young people to generate their own ideas and approaches to interventions, recognizing that young women are best placed to understand their gendered world, identify how to transform their lives, and the delivery mechanisms to do so. Such an argument resonates with other approaches such as developing youth “counter‐publics” 64 and emancipatory community mobilization 65. Re‐envisaging intervention development, however, will require sustained commitment from government, donors and funders to realize its potential. Without this commitment, we run the risk of continuing to miss the mark on preventing IPV and HIV risk for southern Africa's young women.

## Competing interests

The authors have no conflict of interests to declare.

## Authors’ contributions

JM and AG conceptualized the commentary, and JM wrote the first draft AG, SW, MS, JS and LS all commented on and provided intellectual contributions to multiple drafts of the paper.

## Author information

JM is a Lecturer in Global Health at the University College London (UCL) and currently leads a programme of research on intimate partner violence (IPV) in high‐prevalence settings using participatory research approaches. Her research includes the methodological development of participatory approaches for trials of IPV interventions, and the theoretical development of a “distributed” approach to the agency of women and girls in responding to IPV in the Peruvian Amazon, Afghanistan, India and Rwanda. SW is the Capacity Development Manager working on the global programme What Works to Prevent Violence Against Women and Girls (VAWG), and Co‐Investigator of the Stepping Stones and Creating Futures intervention trial, working with young women and men from urban informal settlements in South Africa. Her work involves supporting southern researchers and practitioners to adapt, implement and critically reflect upon interventions to reduce VAWG. She is also completing a PhD reflecting on the role of agency in their experiences and decision‐making, as well as pathways to change. MS holds a Faculty position at the Africa Health Research Institute (AHRI), KwaZulu‐Natal South Africa, leading a programme of HIV prevention in adolescents and youth. The reflections in this Commentary are based in part on her work in evaluating the impact of programmatic scale up of combination prevention for adolescent girls and young women and using this evidence with young people to co‐develop new approaches to improve uptake and retention in combination prevention intervention, such as peer navigation, the use of peers or social networks to deliver HIV self‐testing for linkage to PrEP, and exploring the role of digital health to de‐centralize care. JS is Professor of Anthropology and Health at the London School of Hygiene and Tropical Medicine and faculty lead for Social Science and Research Ethics at the Africa Health Research Institute, KwaZulu‐Natal, South Africa. With others at AHRI she has been working to build a portfolio of research and public engagement activities with and for young people. LS was a founder member and driver on the Coalition for Children affected by AIDS, JLICA and Know Violence. Her research has explored risk and resilience in families infected and affected by HIV. She has been involved in a large number of studies exploring psychosocial provision for children and youth, examining mental health and evaluating interventions in trials in Lesotho, South Africa, Zambia, Malawi and Zimbabwe. She is currently co‐director of the new Accelerate Hub which will be looking at synergies for prevention for adolescents in Africa with Prof Lucie Cluver and Dr Chris Desmond. AG is a Senior Specialist Scientist at the South African Medical Research Council. He is the PI of the Stepping Stones and Creating Futures intervention trial, working with young women and men from urban informal settlements in South Africa. He has a particular interest in understanding how interventions are delivered, and how young people draw on interventions to make changes in their lives.
